# The Effect of the Time Interval Between Radiation and Hyperthermia on Clinical Outcome in 400 Locally Advanced Cervical Carcinoma Patients

**DOI:** 10.3389/fonc.2019.00134

**Published:** 2019-03-08

**Authors:** M. Kroesen, H. T. Mulder, J. M. L. van Holthe, A. A. Aangeenbrug, J. W. M. Mens, H. C. van Doorn, M. M. Paulides, E. Oomen-de Hoop, R. M. Vernhout, L. C. Lutgens, G. C. van Rhoon, M. Franckena

**Affiliations:** ^1^Department of Radiation Oncology, Erasmus MC Cancer Institute, Rotterdam, Netherlands; ^2^Department of Obstetrics and Gynaecology, Erasmus MC Cancer Institute, Rotterdam, Netherlands; ^3^Department of Electrical Engineering, Eindhoven University of Technology, Eindhoven, Netherlands; ^4^Department of Radiation oncology, University Medical Centre Maastricht (MAASTRO), Maastricht, Netherlands

**Keywords:** hyperthermia, cervical cancer, radiotherapy, fractionation, clinical practical procedures, schedulability

## Abstract

**Background:** Addition of deep hyperthermia to radiotherapy results in improved local control (LC) and overall survival compared to radiotherapy alone in cervical carcinoma patients. Based on preclinical data, the time interval between radiotherapy, and hyperthermia is expected to influence treatment outcome. Clinical studies addressing the effect of time interval are sparse. The repercussions for clinical applications are substantial, as the time between radiotherapy and hyperthermia should be kept as short as possible. In this study, we therefore investigated the effect of the time interval between radiotherapy and hyperthermia on treatment outcome.

**Methods:** We analyzed all primary cervical carcinoma patients treated between 1996 and 2016 with thermoradiotherapy at our institute. Data on patients, tumors and treatments were collected, including the thermal dose parameters TRISE and CEM43T90. Follow-up data on tumor status and survival as well as late toxicity were collected. Data was analyzed using Cox proportional hazards analysis and Kaplan Meier analysis.

**Results:** 400 patients were included. Kaplan Meier and univariate Cox analysis showed no effect of the time interval (range 30–230 min) on any clinical outcome measure. Besides known prognostic factors, thermal dose parameters TRISE and CEM43T90 had a significant effect on LC. In multivariate analysis, the thermal dose parameter TRISE (HR 0.649; 95% CI 0.501–0.840) and the use of image guided brachytherapy (HR 0.432; 95% CI 0.214–0.972), but not the time interval, were significant predictors of LC and disease specific survival.

**Conclusions:** The time interval between radiotherapy and hyperthermia, up to 4 h, has no effect on clinical outcome. These results are re-ensuring for our current practice of delivering hyperthermia within maximal 4 h after radiotherapy.

## Introduction

Cervical carcinoma is the sixth most common cancer in women in The Netherlands and the fourth most common cancer in women worldwide(1). Local control (LC) is a prerequisite for definitive cure and contributes highly to patient survival (2). For women with locally advanced cervical cancer, radiotherapy combined with cisplatin-based chemotherapy is the standard treatment (3). In patients unfit for chemotherapy or patients with large FIGO IIB or higher FIGO stage disease, radiotherapy can also be combined with hyperthermia -also called thermoradiotherapy-, instead of chemotherapy. In the latter patients, the addition of hyperthermia to radiotherapy results in comparable outcome compared to chemoradiotherapy in terms of LC and overall survival (OS), while treatment-related toxicity is not increased (4–7). A direct clinical comparison between thermo- and chemoradiotherapy is lacking to date. Nevertheless, a recent network meta-analysis by Datta et al showed that both thermo- as well as chemoradiotherapy were the modalities with the best comprehensive impact on clinical endpoints in cervical cancer patients(8).

At our institute patients are treated standard with thermoradiotherapy in case of a locally advanced (FIGO IIB and higher) tumor or when patients are unfit for or refuse Cisplatin chemotherapy. Induction chemotherapy is indicated in case the tumor is 6 cm or more, lymph node metastases are 2 cm or larger and in case of positive para-aortic lymph nodes. If induction chemotherapy is applied, local treatment consists of thermoradiotherapy, which is given sequentially to the chemotherapy.

Hyperthermia, defined as an elevation of tissue temperature in the range of 40–44 degrees Celsius, is a potent sensitizer of radiotherapy (9, 10). The biological mechanisms of hyperthermia in combination with radiotherapy are diverse and include increased oxygenation, induction of direct cell death and immune stimulation (11, 12). In addition, hyperthermia inhibits the repair of DNA double strand breaks, which are the main inducers of tumor cell death following ionizing radiation (13, 14).

The sensitizing effect of hyperthermia in radiotherapy is determined by the thermal dose, which is the net result of the temperature rise in the target area and the duration of heating (15, 16). Two clinical thermal dose parameters are often investigated are: the CEM43T90 and the linearized TRISE (15). We have previously shown that CEM43T90 and TRISE are independent predictors for LC and Disease Specific Survival (DSS) in a large cohort of cervical cancer patients (15).

For practical reasons and a pre-clinically observed improved therapeutic ratio, hyperthermia is usually applied following external beam radiotherapy treatment (17–19). The effect of the time interval between radiotherapy and hyperthermia on clinical outcome is subject of active investigation. Preclinical evidence suggests a synergistic effect of hyperthermia with radiation when administered within 4 h, with an enhanced effect using shorter time intervals (17, 20). However, the size and relevance of this effect in the clinical setting has not been investigated thoroughly (21, 22). At our institution the hyperthermia treatment is applied within 4 h after the external beam fraction. The waiting time within these 4 h, though, is completely random to date, depending on machine and personnel availability.

Recently, a retrospective analysis showed a significant association between a short time interval and a poor LC and OS (23). Confirmation of this relation would have great impact: all centers treating cervical cancer patients with thermoradiotherapy should reduce the time interval. Therefore, the aim of our study was to analyze the effect of time interval between radiotherapy and hyperthermia on patient outcome in larger cohort of patients with cervical carcinoma cancer.

## Methods

### Patient Population

The research protocol for this investigation was approved by the medical ethics committee of Erasmus MC Cancer Institute, Rotterdam, the Netherlands (MEC-2018-1081). Patients included were diagnosed with primary cervical cancer and treated with curative intent using thermoradiotherapy at our institute between August 1996 and December 2016. Excluded were patients receiving concurrent chemotherapy, fewer than four of the five intended hyperthermia sessions and patients receiving radiotherapy at other institutes because of a lack of data on follow-up. All patients had a histologically confirmed cervical carcinoma and were staged by the International Federation of Gynecology and Obstetrics (FIGO) clinical staging system, including investigation under general anesthesia with cystoscopy and lymph node staging using CT and/or MRI and/or PET-CT. Indications for primary thermoradiotherapy at Erasmus MC Cancer Institute are locally advanced tumors: “lateral” FIGO IIB (>50% parametrial invasion), IIIA, IIIB, and IVA, patients unfit or refusing platinum-based chemotherapy and patients receiving induction chemotherapy. Indications for induction chemotherapy include primary tumor size of 6 cm or larger, pelvic lymph node metastases of 2 cm or larger or para-aortic lymph node metastases (24). Induction chemotherapy consisted of 6-weekly cycles of cisplatin/taxol chemotherapy, with clinical assessment after 3 and 6 cycles.

### Radiotherapy Treatment

Radiotherapy treatment consisted of daily EBRT 23 × 2 Gy for pelvic fields or 28 × 1.8 Gy if the para-aortal region was included in the field. EBRT was delivered using 3D conformal techniques until 2011, after which intensity modulated radiotherapy (IMRT) was gradually introduced. From 2014 onwards VMAT was the preferred technique, because of better conformity and faster treatment times. From 2011 onwards a plan-of-the day protocol was introduced (25). A brachytherapy boost was delivered using a high-dose rate iridium source using 2D planning to point A, with a dose of 2 × 8.5 Gy. From the end of 2012 onwards, image (MRI) guided brachytherapy (IGBT) was applied with a combined interstitial and intracavitary approach, delivering 3 or 4 fractions of 7 Gy or higher to the high-risk CTV (26).

### Hyperthermia Treatment

Hyperthermia (HT) was delivered after radiation therapy, a total of five times during the 5–6 weeks of EBRT. The BSD-2000 system was used (Pyrexar Medical Corporation, Salt Lake City, Utah, USA) for all HT treatments, with the Sigma-60 or Sigma-eye applicator selection depending on the patients' size. Intraluminal thermometry was performed by placing thermometers in rectum, bladder and vagina for all patients. Until July 2012 thermometry was performed by using Bowman probes (Pyrexar Medical Corporation, Salt Lake City, Utah, USA) combined with thermal mapping every 5 min during treatment with a step size of 1 cm and a maximum length of 14 cm. Hereafter temperature measurement was continuous using multi-sensor fiber optic temperature probes (FISO Technologies Inc., Québec, QC, Canada) (4–8 sensor distance between sensors 2 cm). Both systems fulfill the quality assurance guidelines of European Society for Hyperthermic Oncology, accuracy of ±0.2°C (27, 28). Of 90 min scheduled treatment time, the first 30 min are used to warm up to intraluminal temperatures of ≥40°C. Then treatment was continued for 60 min aiming for maximum intraluminal temperatures depending on patient tolerance.

### Collection of Patient and Follow-Up Data

The following patient characteristics were extracted from the patient's files: age at diagnosis, histology, FIGO stage and lymph node status. Local FIGO stage was noted. Seven patients presenting with limited distant metastases, but treated with curative intent, were recorded as FIGO IVB. Induction chemotherapy was noted. Radiotherapy and HT treatment characteristics were extracted from treatment planning and other recording systems.

Pelvic recurrence, distant recurrence, survival status, date and cause of death, as well as late toxicity data were extracted and/or retrieved from patient records, referring hospitals, general physicians and the civil registry. Late toxicity, occurring and/or persisting after 6 months following treatment, was scored according to CTCAE v4.0. Only high-grade (equal or higher than grade 3) toxicities were extracted, as these events are clinically most relevant and most reliably extracted retrospectively.

### Hyperthermia Treatment Parameters

Hyperthermia treatment characteristics collected were the number of hyperthermia sessions, CEM43T90, TRISE and treatment duration. The CEM43T90 is the mean cumulative equivalent minutes of T90 (temperature reached in at least 90% of measurement locations) at 43°C. TRISE is a thermal dose parameter based on the temperature exceeded by 50% of measurement sites and duration of heating (15, 29).

The time interval between EBRT and HT was defined as the time (in minutes) between the first beam-on of the radiotherapy treatment and the start of the heating (power on HT device). As multiple HT treatments are delivered over the course of treatment, the mean time interval between HT and EBRT treatment over all hyperthermia treatments was calculated. In patients in which more than 50% of the time intervals could not be reconstructed, the time interval was noted as missing.

### Statistical Analysis

LC, disease free survival (DFS), DSS and OS were calculated from start date of radiotherapy until event. LC was noted as “failed” when a physician diagnosed a local recurrence either clinically or with imaging (CT/MRI). For DFS, an event was defined as the occurrence of either local or distant recurrence. Patients were censored for local or distant control after the last visit of any physician specifically examining for recurrent disease. For DSS and OS, patients without an event were censored on the day the civil registry was consulted. LC, DFS, DSS, OS were analyzed using the Kaplan–Meier method and statistical differences between groups were determined using the log-rank test. A *p*-value of ≤ 0.05 was considered statistically significant. For KM analyses per time period, the cohort was divided into 5-year periods. Differences in continuous factors between groups were analyzed using the Mann-Whitney U test. Differences in categorical factors between groups were analyzed using the Chi-Square of Fisher's exact test. Hazard ratio (HR) and 95% confidence interval (CI) for various covariates were obtained by univariate Cox proportional hazards analyses. For categorical values, all categories were compared to the index category. In case of small numbers of patients per category of a categorical variable, combinations were made where statistically possible and clinically reasonable to prevent too small subcategories in the analyses. Covariates were taken into multivariable analysis based on clinical experience and a *p*-value ≤ 0.20 in univariate analysis. A backward selection procedure was applied with *p* < 0.05 as a threshold to find the combination of factors that have independent prognostic value. All analyses were performed using IBM SPSS statistics version 24.0 software package (SPSS Inc., Chicago, IL, USA).

## Results

### General Characteristics of the Cohort

Four hundred patients were included in the analysis. General characteristics of the entire cohort are listed in Table 1. 40 (10.0%) patients had an adenocarcinoma and 342 (85.5%) had a squamous cell carcinoma. Eighteen patients (4.5%) had another histology, but were primarily diagnosed as having a cervix uterus origin. The median time interval between start of radiotherapy and start of hyperthermia was 74 min (interquartile range (IQR) 62–94 min) (Table 1). Median follow-up for local and distant recurrence was 57 months (IQR 31–81 months). Median follow-up for survival was 106 months (IQR 52–160 months). There were 113 cases with a local recurrence out of 175 cases with any recurrence. There were 151 disease specific deaths out of 218 total deaths.

**Table 1:** General characteristics of the cohort.

**Characteristic**	**Categories**	**Value**
**PATIENT/TUMOR CHARACTERISTICS**		
Age (years)		55 (IQR 44.0 – 68.8)
Histology	Adeno	40 (10.0%)
	Squamous	342 (85.5%)
	Other	18 (4.5%)
FIGO stage	IB	46 (11.5%)
	IIA	15 (3.8%)
	IIB	174 (43.5%)
	IIIA	18 (4.5%)
	IIIB	102 (25.5%)
	IVA	38 (9.5%)
	IVB	7 (1.8%)
Lymphadenopathy	Negative	197 (49.3%)
	Iliac	139 (34.8%)
	PAO	61 (15.3%)
	Missing	3 (0.8%)
Induction chemotherapy	No	299 (74.8%)
	Yes	101 (25.3%)
**RADIATION THERAPY CHARACTERISTICS**		
Radiation technique	3DCRT	308 (77.0%)
	IMRT	50 (12.5%)
	VMAT	42 (10.5%)
Radiation field	Pelvic	287 (71.8%)
	Pelvic + PAO	113 (28.3%)
Brachytherapy use	No	30 (7.5%)
	Yes	369 (92.3%)
	Missing	1 (0.3%)
Image (MRI) guided brachytherapy	No	334 (83.5%)
	Yes	66 (16.5)
**HYPERTHERMIA TREATMENT CHARACTERISTICS**		
N of treatments	2	10 (2.5%)
	3	11 (2.8%)
	4	50 (12.5%)
	5	329 (82.3%)
Cumulative TRISE (degrees Celsius)		3.46 (2.93-3.86)
	Missing	27 (6.8%)
Cumulative CEM43T90(minutes)		3.40 (IQR 1.89–5.83)
	Missing	27 (6.8%)
Treatment duration (minutes)		90.0 (IQR 88.0-90.0)
	Missing	27 (6.8%)
Mean time interval (minutes)		74.0 (IQR 62.0-94.0)
	Missing	8 (2%)

One time interval could not be reconstructed in 10.0% and two time intervals in 2.0% of patients. In these cases, the mean time interval was calculated using the available interval times. In 8 cases more than 50% of the potential time intervals could not be reconstructed. Temperature data were system-lost in 27 cases. Hence, 373 cases were included in the analysis of thermal dose and 392 for the time interval.

### Kaplan-Meier Analysis of the Effect of Time Interval and Thermal Dose

**Figure 1:**
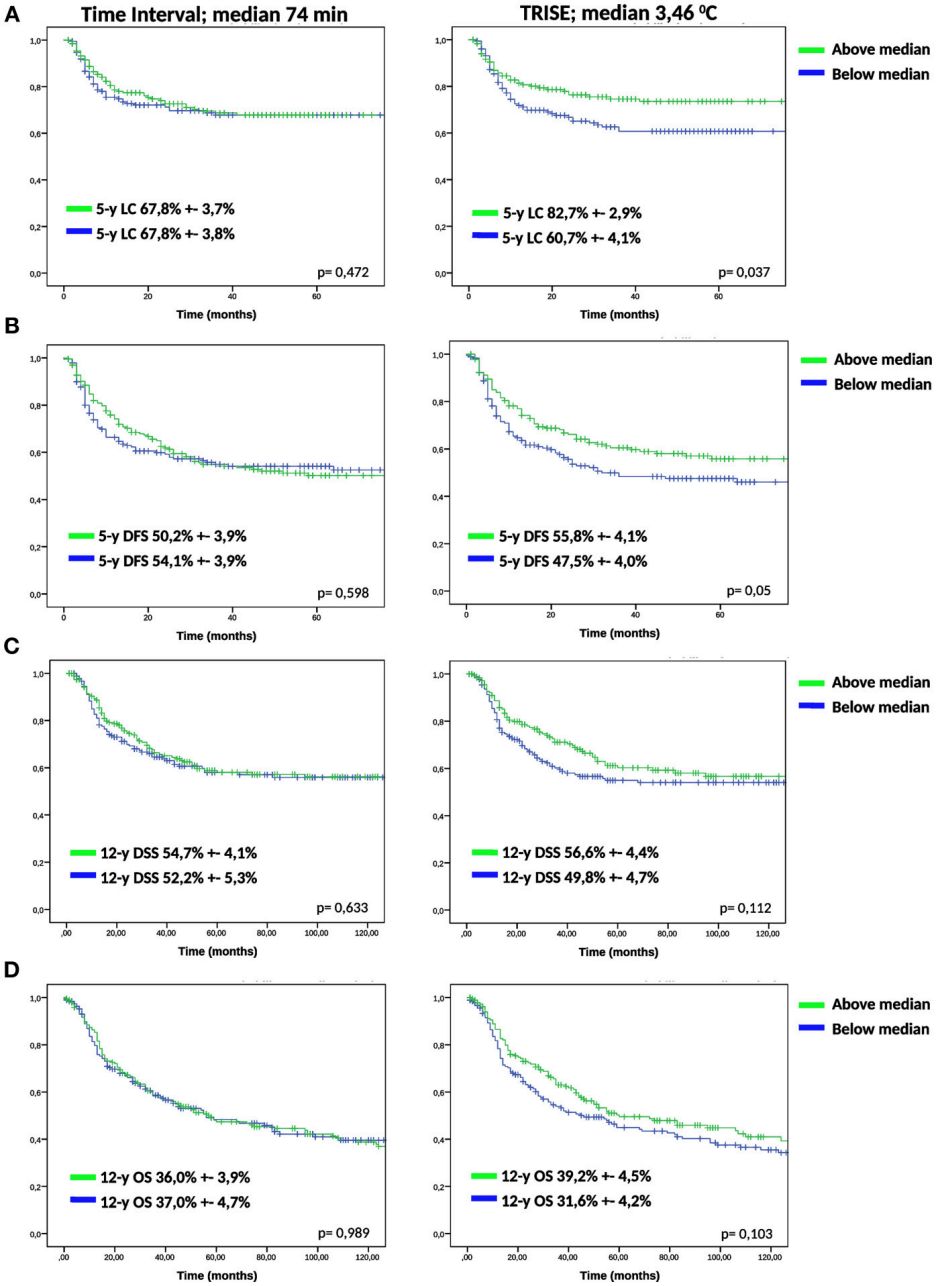
KM analysis of low and high time interval and TRISE. KM-curves for low and high time interval and TRISE for LC **(A)**, DFS **(B)**, DSS **(C)**, and OS **(D)** were compared using log-rank test.

To determine the effect of time interval and thermal dose on clinical outcome, we divided the cohort over the median of the time interval (74 min), TRISE (3.46°C) and CEM43T90 (3.40 min). For the time interval no effect was observed for any of the outcome measures (Figure 1). Figure 1 shows that a higher thermal dose, expressed as TRISE, corresponded to improved outcome for all outcome measures. This effect was statistically significant for LC and DFS. For the thermal dose parameter CEM43T90, KM curves for LC and DFS showed no significant differences, see Supplementary Figure 1.

**Figure 2:**
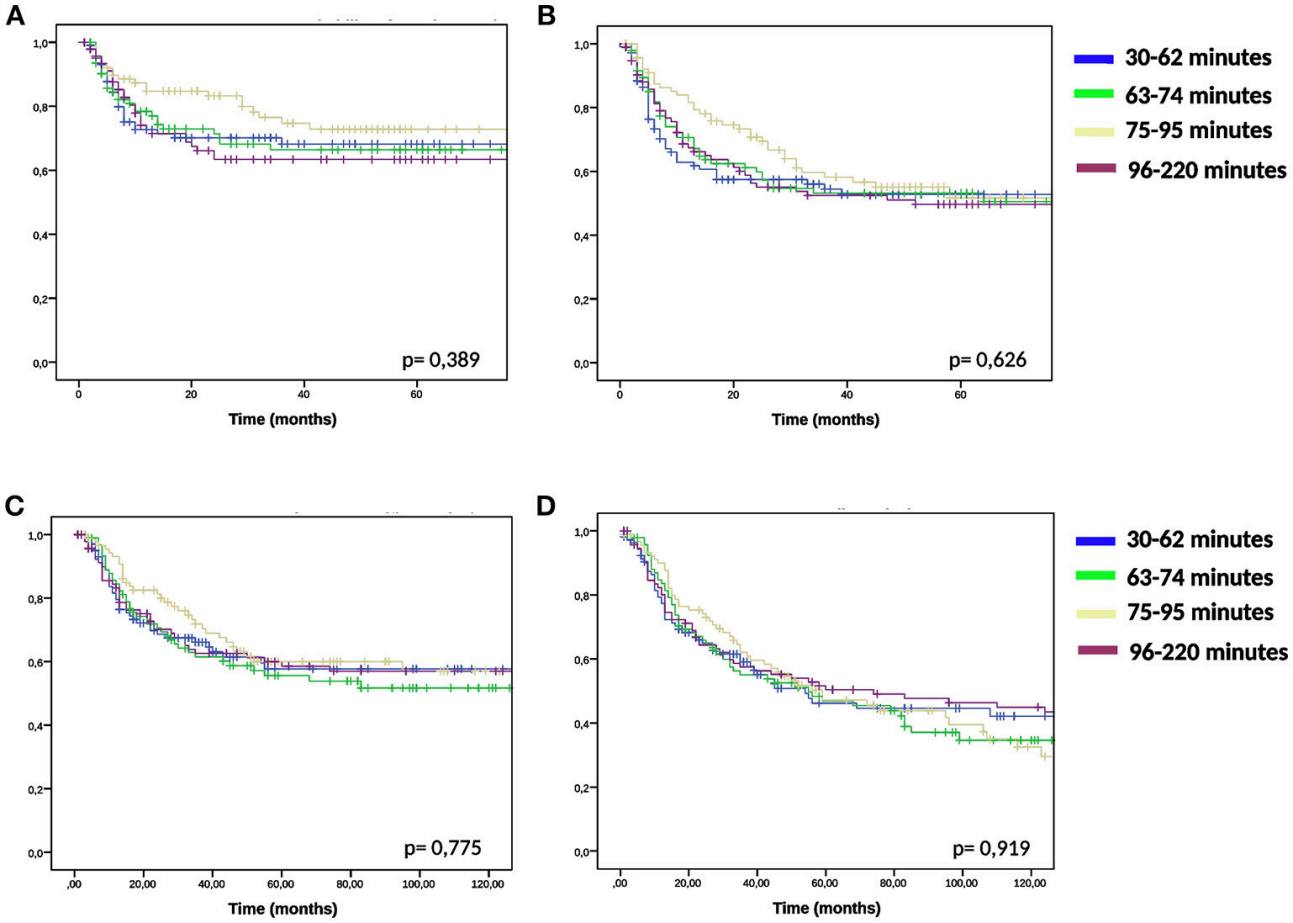
KM analysis of quartile time interval groups. KM-curves for quartiles of the time interval and for LC**(A)**, DFS **(B)**, DSS **(C)**, and OS **(D)** were compared using log-rank test.

We compared the groups higher and lower than median time interval and TRISE for equal distribution of other prognostic factors (Table 2). Patients in the high TRISE group were significantly older and had more HT treatments (Table 2). In the low time interval group significantly more patients were diagnosed with para-aortic lymphadenopathy (20.8 vs. 10.2%) compared to the high time interval group, more patients were treated with induction chemotherapy (34.4 vs. 17.0%, *p* ≤ 0,001) and more with VMAT (16.7 vs. 5.0%, *p* = 0.001) and IGBT (20.8 vs. 13.0%, *p* = 0.038). These differences are explained by the length of the time interval, which became shorter over time (Supplementary Figure 2). KM analyses performed in four different time periods showed no differences in LC between high and low time interval patient groups (Supplementary Figure 3), as well as in DFS, DSS and OS (data not shown). In order to determine the effect of very short or very long time intervals, cases were divided over the quartiles of the time interval. Again no differences were observed for LC, DFS, DSS and OS between the four time interval groups (Figure 2).

**Table 2:** Comparison of low and high TRISE and low and high time interval groups.

**Characteristic**	**Category**	**Low TRISE** **(*n* =186)**	**High TRISE** **(*n* = 187)**	***p*-value**	**Low time** **interval (*n*= 192)**	**High time interval** **(*n*= 200)**	***p*-value**
Age (years)		52.0 (IQR 39.8–66.0)	59.0 (IQR 49.0–72.0)	< 0.001	53.5 (IQR 44.0–66.0)	57.0 (IQR 44.0–71.0)	0.118
Histology				0.229			0.504
	Adeno	15 (8.1%)	23 (12.3%)		22 (11.5%)	17 (8.5%)	
	Squamous	164 (88.2%)	153 (81.8%)		160 (83.3%)	175 (87.5%)	
	Other	7 (3.8%)	11 (5.9%)		10 (5.2%)	8 (4.0%)	
FIGO stage				0.093			0.513
	IB	27 (14.5%)	14 (7.5%)		27 (14.1%)	17 (8.5%)	
	IIA	6 (3.2%)	7 (3.7%)		6 (3.1%)	9 (4.5%)	
	IIB	68 (36.6%)	93 (49.7%)		86 (44.8%)	87 (43.5%)	
	IIIA	9 (4.8%)	9 (4.8%)		10 (5.2%)	8 (4.0%)	
	IIIB	48 (25.8%)	47 (25.1%)		42 (21.9%)	55 (27.5%)	
	IVA	24 (12.9%)	14 (7.5%)		17 (8.9%)	21 (10.5%)	
	IVB	4 (2.2%)	3 (1.6%)		4 (2.1%)	3 (1.5%)	
Lymphadenopathy				0.366			0.003
	Negative	98 (53.3%)	86 (46.2%)		81 (42.2%)	111 (56.3%)	
	Iliac	61 (33.2%)	68 (36.6%)		71 (37.0%)	66 (33.5%)	
	PAO	25 (13.6%)	32 (17.2%)		40 (20.8%)	20 (10.2%)	
Induction				0.576			< 0.001
chemotherapy	No	140 (75.3%)	136 (72.7%)		126 (65.6%)	166 (83.0%)	
	Yes	46 (24.7%)	51 (27.3%)		66 (34.4%)	34 (17.0%)	
Radiation technique				0.283			0.001
	3DCRT	146 (78.5%)	135 (72.2%)		137 (71.4%)	163 (81.5%)	
	IMRT	20 (10.8%)	30 (16.0%)		23 (12.0%)	27 (13.5%)	
	VMAT	20 (10.8%)	22 (11.8%)		32 (16.7%)	10 (5.0%)	
Radiation field				0.148			0.978
	Pelvic	138 (74.2%)	126 (67.4%)		138 (71.9%)	144 (72.0%)	
	Pelvic + PAO	48 (25.8%)	61 (32.6%)		54 (28.1%)	56 (28.0%)	
IGBT use				0.805			0.038
	No	154 (82.8%)	153 (81.8%)		152 (79.2%)	174 (87.0%)	
	Yes	32 (17.2%)	34 (18.2%)		40 (20.8%)	26 (13.0%)	
No of treatments				< 0.001			0.253
	2	7 (3.8%)	0 (0%)		3 (1.6%)	7 (3.5%)	
	3	9 (4.8%)	0 (0%)		3 (1.6%)	6 (3.0%)	
	4	42 (22.6%)	5 (2.7%)		25 (13.0%)	23 (11.5%)	
	5	128 (68.8%)	182 (97.3%)		161 (83.9%)	164 (82.0%)	
Cumulative CEM43T90				< 0.001			0.764
(minutes)		2.05 (IQR 1.20–3.01)	5.69 (IQR 3.9–7.64)		3.55 (IQR 2.03–5.61)	3.14 (IQR 1.66–6.2)	
Cumulative TRISE							0.656
(degrees Celsius)					3.44 (IQR 2.93–3.87)	3.51 (IQR 2.93–3.88)	
Time interval (minutes)				0.347			
		72.5 (IQR 62.0–92.5)	75.0 (IQR 62.0–95.5)				

*Comparison of patients divided over the median TRISE and time interval*.

### Univariate and Multivariate Cox Proportional Hazard Analysis

Using univariate Cox proportional hazards regression analysis the influence of known prognostic factors, time interval and thermal dose on LC, DFS, DSS, and OS was determined (Table 3). The time interval showed no effect on LC, DFS, DSS, and OS with Hazard Ratios of 1. Histology (squamous cell vs. adenocarcinoma; HR 0.48; 95% CI 0.29–0.81), FIGO stage (FIGO IIIA + IIIB vs. FIGO IB; HR 2.72; 95% CI 1.37–5.39), CEM43T90 (HR 0.93; 95% CI 0.87–0.99), TRISE (HR 0.67; 95% CI 0.52–0.86) and IGBT (HR 0.41; 95% CI 0.21–0.82) showed a significant effect on LC in univariate analysis. FIGO stage, TRISE, and IGBT also showed a significant effect on DFS, DSS, and OS.

**Table 3:** Univariate and multivariate Cox regression analysis.

**Univariate** **analysis**	**Unit variable increase**	**LC**		**DFS**		**DSS**		**OS**	
		**HR (95% CI)**	***p*-value**	**HR (95% CI)**	***p*-value**	**HR (95% CI)**	***p*-value**	**HR (95% CI)**	***p*-value**
Age	years	1.00 (0.98–1.01)	0.547	1.00 (0.99–1.01)	0.967	1.00 (0.99–1.01)	0.930	1.02 (1.01–1.03)	< 0.001
Histology			0.016		0.014		0.199		0.077
	squamous vs. adeno	0.48 (0.29–0.81)	0.006	0.54 (0.3–0.82)	0.004	0.65 (0.41–1.05)	0.077	0.63 (0.42–0.94)	0.025
	other vs. adeno	0.77 (0.32–1.86)	0.559	0.72 (0.34–1.55)		0.77 (0.34–1.76)	0.541	0.74 (0.37–1.49)	0.403
FIGO stage			< 0.001		< 0.001		< 0.001		< 0.001
	IIA+ IIB vs. IB	1.15 (0.58–2.28)	0.695	1.32 (0.76–2.31)	0.322	1.34 (0.72–2.50)	0.349	1.70 (0.99–2.94)	0.057
	IIIA + IIIB vs. IB	2.72 (1.38–5.39)	0.004	2.63 (1.50–4.60)	0.001	2.98 (1.60–5.53)	0.001	3.36 (1.94–5.84)	< 0.001
	IVA + IVB vs. IB	1.38 (0.58–3.33)	0.470	1.69 (0.85–3.35)	0.135	2.10 (1.00–4.40)	0.050	2.81 (1.49–5.29)	0.001
Lymphadenopathy			0.201		0.360		0.268		0.587
	iliac vs. negative	1.38 (0.93–2.06)	0.114	1.22 (0.87–1.69)	0.249	1.14 (0.79–1.64)	0.482	0.85 (0.63–1.16)	0.853
	PAO vs. negative	0.92 (0.51–1.66)	0.768	1.29 (0.85–1.97)	0.231	1.43 (0.93–2.21)	0.106	0.97 (0.66–1.42)	0.966
Time interval	minutes	1.00 (0.99–1.01)	0.565	1.00 (0.99–1.01)	0.597	1.00 (0.99–1.01)	0.655	1.00 (0.99–1.00)	0.855
CEM43T90	minutes	0.93 (0.87–1.00)	0.035	0.95 (0.90–1.00)	0.053	0.96 (0.91–1.02)	0.155	1.01 (0.97–1.01)	0.491
TRISE	degrees Celsius	0.67 (0.52–0.86)	0.002	0.75 (0.60–0.92)	0.006	0.73 (0.58–0.91)	0.006	0.80 (0.65–0.96)	0.016
IGBT use	yes vs. no	0.41 (0.21–0.82)	0.011	0.56 (0.34–0.91)	0.020	0.38 (0.20–0.73)	0.003	0.43 (0.25–0.74)	0.002
**MULTIVARIATE ANALYSIS**									
Histology			0.010		0.024		n.s.		0.071
	squamous vs. adeno	0.45 (0.26–0.78)	0.004	0.53 (0.33–0.85)	0.008			0.603 (0.39–0.94)	0.024
	other vs. adeno	0.65 (0.29–1.46)	0.587	0.74 (0.34–1.62)	0.454			0.75 (0.36–1.54)	0.428
FIGO stage			0.010		0.002		< 0.001		< 0.001
	IIA+ IIB vs. IB	1.50 (0.72–3.11)	0.278	1.69 (0.92–3.10)	0.090	1.82 (0.92–3.61)	0.086	1.98 (1.10–3.58)	0.024
	IIIA + IIIB vs. IB	2.80 (1.34–5.84)	0.006	2.86 (1.55–5.27)	0.001	3.54 (1.78–7.07)	< 0.001	3.22 (1.77–5.85)	< 0.001
	IVA + IVB vs. IB	1.74 (0.70–4.30)	0.233	2.03 (0.98–4.17)	0.055	2.45 (1.11–5.37)	0.026	3.38 (1.74–6.60)	< 0.001
Lymphadenopathy			0.031		0.051		0.011		n.s.
	iliac vs. negative	1.81 (1.16–2.81)	0.009	1.42 (0.99–2.05)	0.059	1.48 (0.99–2.20)	0.057		
	PAO vs. negative	1.29 (0.70–2.39)	0.419	1.65 (1.05–2.58)	0.031	2.00 (1.25–3.19)	0.004		
TRISE	degrees celsius	0.65 (0.50–0.84)	0.001	0.72 (0.58–0.89)	0.003	0.71 (0.57–0.90)	0.004	0.79 (0.65–0.96)	0.019
IGBT use	yes vs. no	0.43 (0.21–0.97)	0.019	0.58 (0.35–0.96)	0.035	0.41 (0.21–0.78)	0.007	0.46 (0.27–0.81)	0.006
CEM43T90^*^	minutes	0.92 (0.85–0.99)	0.019	0.94 (0.89–0.99)	0.019	0.94 (0.89–1.00)	0.051		n.s.

*Cox proportional hazard analysis for LC, DFS, DSS, and OS*.

* *In multivariate analysis using CEM43T90, other factors showed similar results compared to results shown for TRISE*.

Multivariate Cox analyses were performed (Table 3). As CEM43T90 and TRISE are both indicators of thermal dose, these factors were introduced separately into the analyses. Histology (squamous cell vs. adenocarcinoma; HR 0.45; 95% CI 0.26–0.78), FIGO stage (FIGO IIIA + IIIB vs. FIGO IB; HR 2.80; 95% CI 1.34–5.84), lymphadenopathy (iliac vs. negative; HR 1.80; 95% CI 1.16–2.81), CEM43T90 (HR 0.92; 95% CI 0.85–0.99), TRISE (HR 0.65; 95% CI 0.50–0.84), and IGBT (HR 0.43; 95% CI 0.21–0.97) showed a significant effect on LC (Table 3). Note that having another histology than adeno- or squamous cell carcinoma did not have a significant effect on any of the clinical endpoints (Table 3). For DSS, independent prognostic factors were FIGO stage, lymphadenopathy, TRISE, and IGBT. Time interval was added to every analysis, but was always removed because of non-significance.

### Effect of Time Interval and Thermal Dose on Late Toxicity

To determine the effect of time interval and thermal dose on toxicity, grade 3 or higher late toxicity was analyzed. The incidence of late toxicity did not differ between low or high TRISE (10.2 vs. 11.2%, respectively, *p* = 0.751) or low or high time interval patients (10.4 vs. 10.5%, respectively, *p* = 0.978) (Table 4).

**Table 4:** Comparison of late toxicity in low and high TRISE and low and high time interval groups.

	**Below median Time Interval**	**Above median Time Interval**	**Total**	***p*-value**
				0.978
No grade 3 or higher toxicity	172 (89.6%)	179 (89.5%)	351 (89.5%)	
Grade 3 or higher toxicity	20 (10.4%)	21 (10.5%)	41 (10.5%)	
Total	192	200	392	
	**Below median TRISE**	**Above median TRISE**	**Total**	***p*****-value**
				0.751
No grade 3 or higher toxicity	167 (89.8%)	166 (88.8%)	333 (89.3%)	
Grade 3 or higher toxicity	19 (10.2%)	21 (11.2%)	40 (10.7%)	
Total	186	187	373	

*Incidence of grade 3 or higher toxicity in patients with low or high time interval and TRISE*.

## Discussion

From this large retrospective cohort study, we conclude that a time interval between radiotherapy and hyperthermia up to 4 h has no effect on clinical outcome. Other known prognostic factors; use of IGBT and the thermal dose did have an effect on clinical outcome.

Hyperthermia is a potent sensitizer of radiotherapy in cancer treatment (6, 11, 17, 18, 30–33). Based on preclinical data, it could be hypothesized that a shorter time interval between radiotherapy and hyperthermia results in improved clinical outcome. This is largely based on the -relatively recent- insight that hyperthermia inhibits DNA repair in combination with the observation that most DNA damage after radiotherapy is repaired within 2–6 h (13, 20, 23). Several preclinical studies have analyzed the influence of the time interval (17, 20, 34). Li et al showed enhanced cell kill efficacy upon a shorter time interval (34). Overgaard et al. using a murine model, showed that at a time interval of 4 h, the normal tissue damage is minimized compared to the tumor damage (17). This study also showed that a shorter time interval increased tumor cell killing, but at the cost of increased normal tissue toxicity. The drawback of these two studies is that the target temperatures used, exceed those generally measured in the clinic. More recently, two cervical carcinoma cell lines were treated with hyperthermia and radiotherapy at different time intervals and target temperatures (20). There was a small increase in cell killing at shorter time intervals, but the effect was less apparent than in previous studies (17, 34). Finally, preclinical data generated at our institute indicate no enhanced effect of a shorter time interval on tumor cell kill (personal communication R. Kanaar, Dept. of Molecular Genetics).

There are few clinical studies which have investigated the clinical relevance of the time interval between radiotherapy and hyperthermia (21, 22). Arcangeli et al showed that skin toxicity was reduced with an interval of 4 h, compared to simultaneous application (21). Lindholm et al compared short and long time intervals in patients with superficial tumors (22). In both studies patient numbers were too small to draw firm conclusions. More recently, in a cohort of 58 primary cervical carcinoma patients, a significant association between a lower than median time interval and improved LC and OS was observed (23). To explain for these different findings, firstly patient characteristics may be different between the two cohorts. In the cohort of Van Leeuwen et al. patients are only indicated for thermoradiotherapy when chemoradiation was not possible due to renal function impairment, co-morbidity or age. Therefore, these patients are potentially more frail compared to the patients in our cohort. Second, it is possible that thermal dose was higher compared to our cohort, leading to a more pronounced biological effect of the time interval in the cohort of Van Leeuwen. Finally, in the study by Van leeuwen, following dichotomization of the time interval for KM analysis, the dichotomized time interval was also used in Cox proportional hazard analysis. Instead, we used the continuous value of the time interval in Cox analysis, as this is more informative and less sensitive to false positive results (35). Future clinical studies, preferably prospective, should provide a more definitive answer to this relevant clinical issue.

Besides the abovementioned inhibition of DNA repair, hyperthermia can also sensitize tumor cells to radiotherapy through various other mechanisms. These mechanisms include increased oxygenation, induction of direct tumor cell death and immune stimulation (12). Little is known about the exact biological and physiological mechanisms that constitute hyperthermia's proven clinical effectiveness. Increased perfusion and thus increased oxygenation can be expected at temperatures ≥39°C and may hold for many hours after treatment (36, 37). The direct hyperthermia-induced cytotoxicity seems to come in play at temperatures of ≥40°C. This effect is short-lived after treatment but results in spatial cooperation as it mainly effects cancer cells that are relatively insensitive to the effects of radiotherapy and chemotherapy (19, 38, 39). Studies investigating the effect of local or loco-regional hyperthermia on the immune system have not yet looked into the time or temperature dependency to our knowledge (40). The relative contribution of these effects to the clinical effect of hyperthermia is unknown. Our finding of a null-effect of the time interval could be interpreted as that DNA repair inhibition is relatively unimportant for the clinical hyperthermia effect. But it could also imply that in patients, it takes longer before the DNA repair inhibition effect disappears, compared to *in vitro* studies.

A limitation of our study is the retrospective setup, with the potential for confounding, data misinterpretation and data loss. The latter could be relevant for the collection of the late toxicity data in our study, as it was not recorded in a standardized manner at Erasmus MC Cancer Institute during the time of inclusion. Therefore, we chose not to include Grade 1–2 late toxicities as these might have been recorded incompletely. Grade 3 or higher toxicities are more likely to be recorded as by definition, a medical intervention is required. As potential miss of toxicity is not related to the studied factors (TRISE, time interval), we do not expect the statistical analysis to be influenced by this potential miss of data.

## Conclusions

In contrast to thermal dose, there is no effect of the time interval between radiotherapy and hyperthermia on clinical outcome in primary cervical carcinoma patients. The clinical consequence of this study is that our current recommendation to apply hyperthermia within 4 h after radiation was and remains valid for cervical carcinoma patients treated with thermoradiotherapy. These finding are also relevant for the, mostly centralized, clinical hyperthermia units, as patients can be re-assured that their outcome is not affected by travel times up to 4 h.

## Data Availability

The datasets generated for this study are available on request to the corresponding author.

## Author Contributions

MK: drafting the article, data analysis, data extraction; HM: data extraction, data analysis, critically revising the article; JvH: data extraction, critically revising the article; AA: data extraction; JM: critically revising the article; HvD: critically revising the article; MP: critically revising the article, data extraction, data analysis; EO: data analysis, critically revising the article; RV: critically revising the article; LL: critically revising the article; GvR: critically revising the article; MF: data extraction, data analysis, critically revising the article.

### Conflict of Interest Statement

GvR reported conflict of interests outside the submitted work: Dr. G. Sennewald Medizintechnik GmbH (Support conference participation), Pyrexar Medical (Research MR-thermometry), Sensius BV (Consultant (unpaid)). MP reported conflict of interests outside the submitted work: Sensius BV (Consultant (unpaid)). The remaining authors declare that the research was conducted in the absence of any commercial or financial relationships that could be construed as a potential conflict of interest.

## Supplementary Material

Supplementary Figure 1 KM analysis of low and high CEM43T90 groups. KM-curves for low and high CEM43T90 for LC **(A)** and DFS **(B)** were compared using log-rank test.

Supplementary Figure 2 Development of the time interval in time. Shown are the different time periods used in subsequent KM-analysis.

Supplementary Figure 3 KM analysis of low and high time interval per time period. KM-curves for low and high time interval for LC were compared using log-rank test.
