# Supplementary figures and images for: The Effect of the Time Interval Between Radiation and Hyperthermia on Clinical Outcome in 400 Locally Advanced Cervical Carcinoma Patients
Source: Front Oncol. 2019 Mar 8;9:134. doi: 10.3389/fonc.2019.00134 (PMC6418024; doi:10.3389/fonc.2019.00134)

**A** CEM43T90; median 3,40 minutes

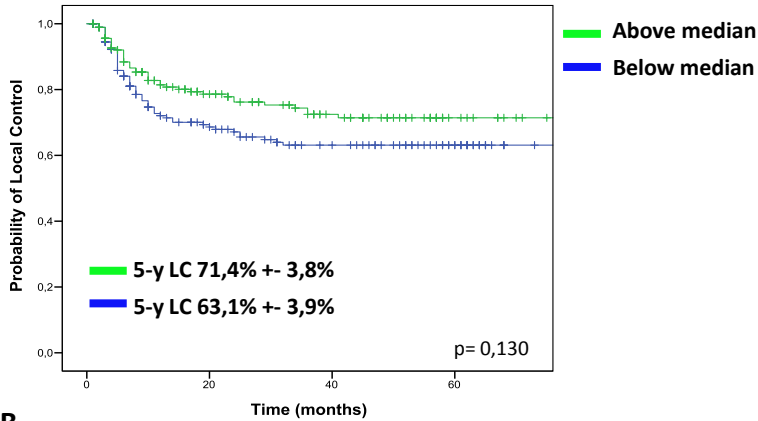

**B**

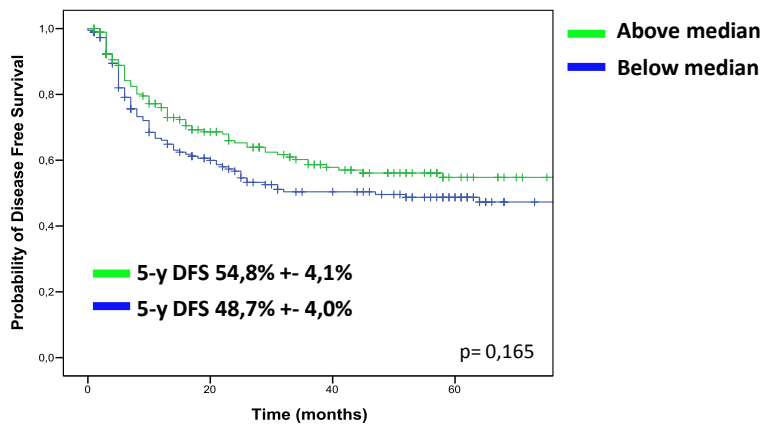

Supplement: Supplementary Figure 1 — KM analysis of low and high CEM43T90 groups. KM-curves for low and high CEM43T90 for LC (A) and DFS (B) were compared using log-rank test. [file Image_1.pdf]

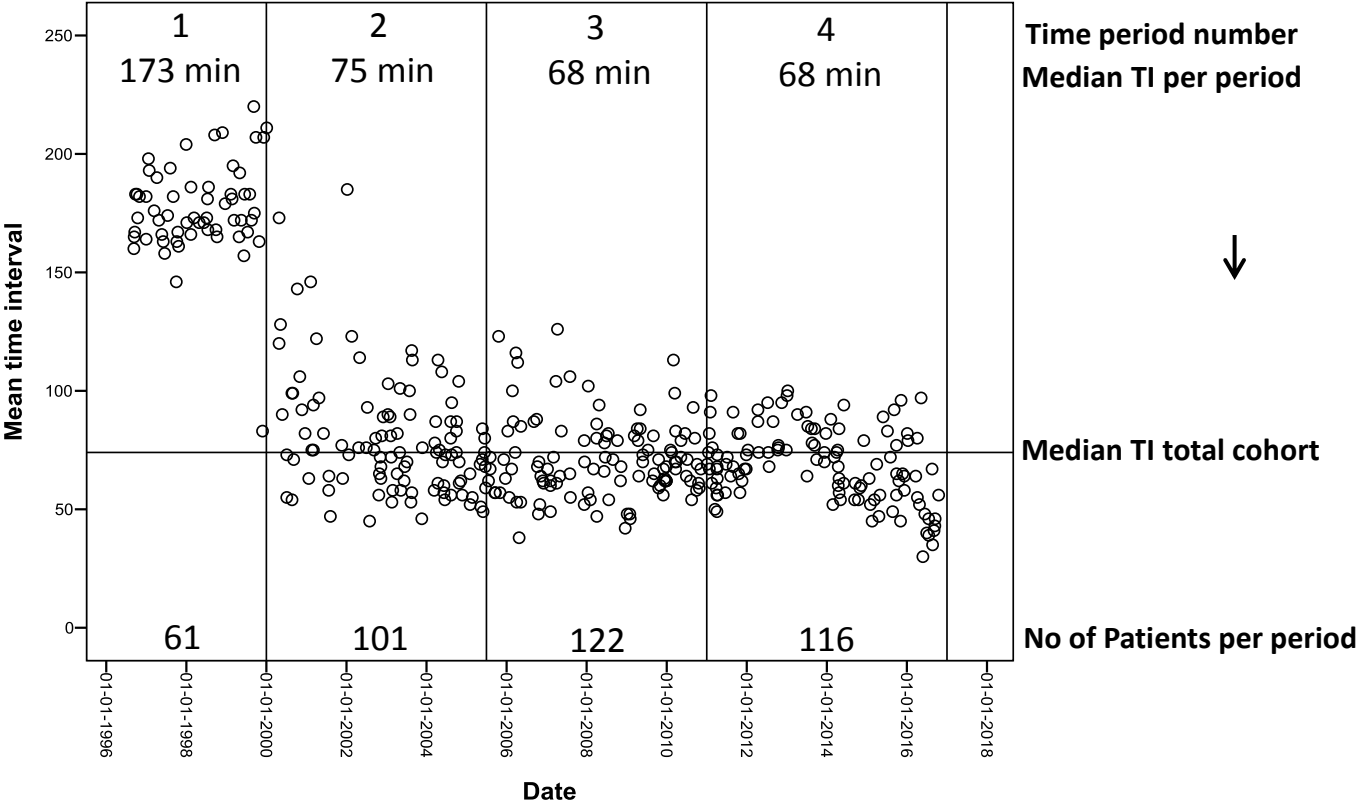

Supplement: Supplementary Figure 2 — Development of the time interval in time. Shown are the different time periods used in subsequent KM-analysis. [file Image_2.pdf]

Time period 1; median 173 min.

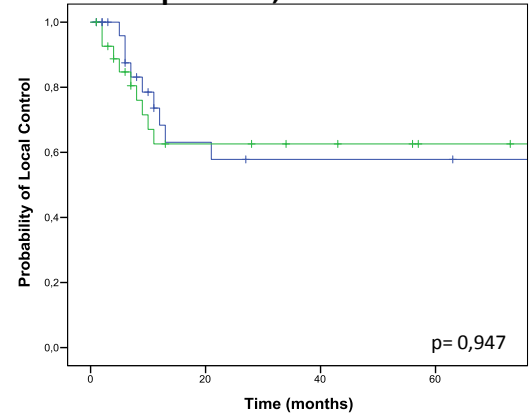

Time period 2; median 75 min.

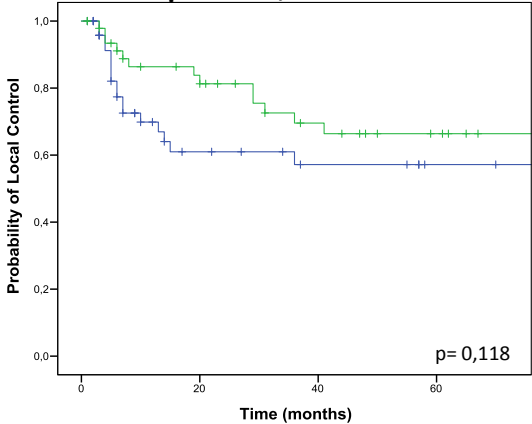

Time period 3; median 68 min.

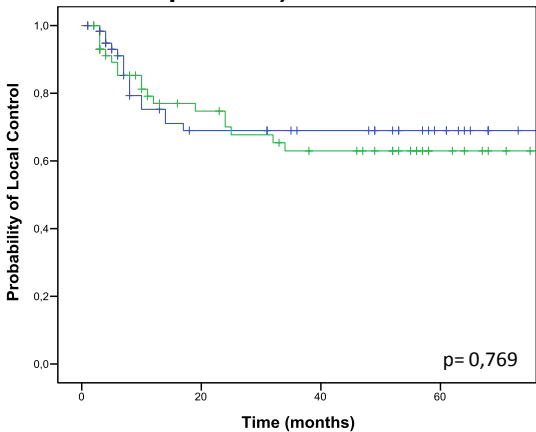

Time period 4; median 68 min.

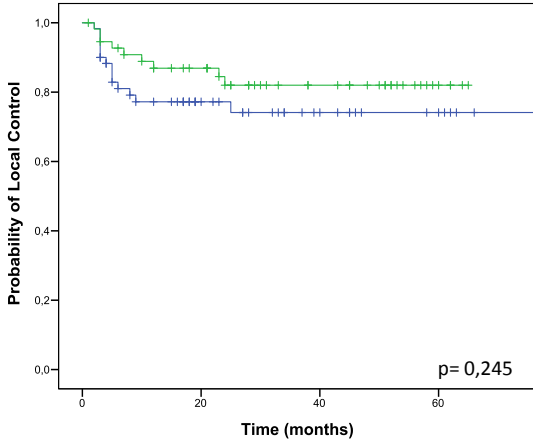

Supplement: Supplementary Figure 3 — KM analysis of low and high time interval per time period. KM-curves for low and high time interval for LC were compared using log-rank test. [file Image_3.pdf]
